# Professional Items

**Published:** 1849-07

**Authors:** 


*Professional Items.*—Among the dentists who have gone to California, so far as we have heard, are Drs. B. B. Brown of St. Louis, Mo., Weems of Franklin, Tenn., W. Ware of Wilmington, North Carolina, Hays of Buffalo, N. Y., and Leech of Baltimore. We hope the golden expectations of each may be fully realized. It is the intention of Dr. Brown, we believe, to abandon the practice of dental surgery for that of general medicine. We are sorry to lose from our own immediate department of the profession, so scientific and skillful a practitioner, but we believe he is influenced in making this change, by the state of his general health.

[*Balt. Ed.*

